# Peripheral Blood Smears in COVID-19: A Comment

**DOI:** 10.4274/tjh.galenos.2020.2020.0704

**Published:** 2021-02-25

**Authors:** Rujittika Mungmunpuntipantip, Viroj Wiwanitkit

**Affiliations:** 1 Private Academic Consultant, Bangkok, Thailand; 2 Joseph Ayobabalola University, Ikeji-Arakeji, Nigeria

**Keywords:** COVID-19, Blood smear, Laboratory

## To the Editor,

We would like to share our ideas on the recently published letter entitled “Peripheral Blood Smear Findings in COVID-19” [1]. Ahnach et al. [1] reported several observations and concluded that “Our preliminary results remain limited and more investigation is required to study the reversibility of these abnormalities and their impact on severity. Compared to all inflammatory biomarkers, observation of blood cells can be a simple alternative for the first triage and early identification of the infection” [1]. We agree that blood cell changes might be different in coronavirus disease-19 (COVID-19) patients with different stages of disease and severity. Focusing on the immunopathogenesis seen in COVID-19, stimulation of lymphocytes might occur and this might be similar to the pathological process seen in dengue. Regarding platelets, thrombocytopenia is a common problem and decreased platelet counts should be seen in peripheral blood smears [2]. Finally, there is another point that Ahnach et al. [1] did not mention. There might also be some underlying diseases or concurrent disorders in COVID-19 patients that could result in blood cell abnormalities. For example, there might be a concurrence between COVID-19 and parasitic infection, or the patient might have an underlying allergic problem related to the change of eosinophils [3,4].
